# NMR chemical shift assignment of *Drosophila* odorant binding protein 44a in complex with 8(Z)-eicosenoic acid

**DOI:** 10.1007/s12104-024-10178-2

**Published:** 2024-06-01

**Authors:** Myriam L. Cotten, Mary R. Starich, Yi He, Jun Yin, Quan Yuan, Nico Tjandra

**Affiliations:** 1 Department of Biochemistry and Biophysics, Oregon State University, Corvallis, OR 97331 USA; 2 Laboratory of Molecular Biophysics, Biochemistry and Biophysics Center, National Heart, Lung, and Blood Institute, National Institutes of Health, Bethesda, MD 20892 USA; 3 Fermentation Facility, Biochemistry and Biophysics Center, National Heart, Lung, and Blood Institute, National Institutes of Health, Bethesda, MD 20892 USA; 4 Dendrite Morphogenesis and Plasticity Unit, National Institute of Neurological Disorders and Stroke, National Institutes of Health, Bethesda, MD 20892 USA

**Keywords:** Odorant binding protein, Fatty acid binding protein, Lipid interaction

## Abstract

The odorant binding protein, OBP44a is one of the most abundant proteins expressed in the brain of the developing fruit fly *Drosophila melanogaster*. Its cellular function has not yet been determined. The OBP family of proteins is well established to recognize hydrophobic molecules. In this study, NMR is employed to structurally characterize OBP44a. NMR chemical shift perturbation measurements confirm that OBP44a binds to fatty acids. Complete assignments of the backbone chemical shifts and secondary chemical shift analysis demonstrate that the apo state of OBP44a is comprised of six α-helices. Upon binding 8(Z)-eicosenoic acid (8(Z)-C20:1), the OBP44a C-terminal region undergoes a conformational change, from unstructured to α-helical. In addition to C-terminal restructuring upon ligand binding, some hydrophobic residues show dramatic chemical shift changes. Surprisingly, several charged residues are also strongly affected by lipid binding. Some of these residues could represent key structural features that OBP44a relies on to perform its cellular function. The NMR chemical shift assignment is the first step towards characterizing the structure of OBP44a and how specific residues might play a role in lipid binding and release. This information will be important in deciphering the biological function of OBP44a during fly brain development.

**Supplementary Information:**

The online version contains supplementary material available at 10.1007/s12104-024-10178-2.

## Biological context

Insects pick up cues from their surroundings that inform them of environmental conditions, availability of food, mating potential and other signals critical to survival (Rihani et al. 2021). These cues typically appear in the form of small, often hydrophobic, chemical compounds. They are detected by sensory organs of the insects, such as the antenna, and must be transported to reach the sensory receptors. Insects have several families of proteins whose role is to transfer these small molecules across the aqueous environment within the insect (Getchell et al. 1984; Rihani et al. 2021).

Odorant binding proteins (OBPs) are one such family of proteins, which were originally identified as carriers for the odorant molecules in insects and vertebrates (Leal 2013; Pelosi et al. 2006; Pelosi and Maida 1995; Steinbrecht 1998; Tegonia et al. 2000; Vogt 2005). They are small soluble proteins that can bind hydrophobic molecules with relatively high affinity and are expressed in essentially all organs. Knowledge of their roles has expanded to demonstrate roles beyond transport of odorant to olfactory receptors. In fact, their function is quite broad, including taste (Scheuermann and Smith 2019; Harada et al. 2008; Matsuo et al. 2007; Li et al. 2019), gut and immune response (Benoit et al. 2017), mating behavior (Swarup et al. 2011), protection against oxidative stress (Benoit et al. 2017; Guo et al. 2021) and even humidity detection (Sun et al. 2018).

OBP44a is a member of this family of protein and one among 52 others that have been identified in the fruit fly *Drosophila melanogaster* (Larter et al. 2016). Interestingly, OBP44a is one of the most abundant proteins expressed during the early development of the Drosophila brain (Yin et al. 2024). The cellular function of this protein is still not known. One of the molecular functions, however, is quite clear. As a member of the OBP family, it is expected to be able to bind hydrophobic ligands (for a review please see (Rihani et al. 2021)). It is tantalizing to suspect that the high level of expression during development points to the role of OBP44a in metabolic, signaling, or homeostatic processes by transporting its ligands intra- and extra-cellularly in the developing larval brain.

To better understand the potential roles of *Drosophila* OBP44a, we have initiated structural and biochemical characterizations of OBP44a. We confirmed that OBP44a can bind several fatty acids and NMR results indicate that the protein has strong affinity towards 8(Z)-eicosenoic acid. NMR assignments for holo OBP44a in complex with 8(Z)-eicosenoic acid and its apo form are reported here. Through secondary chemical shift analysis, the protein is shown to contain six α-helices. A comparison of the NMR data acquired for apo and holo forms of OBP44a allowed for the mapping of a region in the C-terminal domain of the protein that undergoes conformational changes upon fatty acid binding. We propose that this region plays determinant roles in enabling OBP44a to regulate the uptake and release of ligands in the developing brain of the fruit fly.

## Methods and experiments

### Materials

Unless otherwise indicated the reagents were obtained from Sigma Aldrich (St. Louis, MO). The ligand 8(Z)-eicosenoic acid was obtained from Cayman Chemical (Ann Arbor, MI). All stable isotopes were from Cambridge Isotope Laboratories (Andover, MA).

### Sample preparation

The ^15^N and ^13^C, ^15^N isotope labeled *Drosophila* OBP44a samples were prepared using a recently published protocol (He et al. 2023). Of importance is the final purification step involving reverse phase HPLC using a Zorbax C8 preparative column, following FPLC purification and TEV cleavage of the histidine tag of the protein. A linear gradient from 0 to 70% of water and acetonitrile (Fischer Chemical) containing 0.1% TFA was run through the column. The protein eluted at 44% acetonitrile. The protein fraction was frozen and lyophilized on an SP Scientific Benchtop Pro Ominitronics system. This was followed by reconstituting the protein in 0.01 M HCl to neutralize the trifluoroacetate counterions and removing residual TFA through extensive lyophilization. The protein powder was dissolved in water and the pH checked and adjusted to neutral by adding a small amount of 0.1 M NaOH. It was then frozen and lyophilized for storage at -20 °C. This procedure ensures that the purified OBP44a is in the apo state and free of any bound hydrophobic moieties.

All NMR samples were prepared by dissolving an appropriate amount of the protein powder in 20 mM potassium phosphate buffer at pH 6.62 with 0.5 mM EDTA. The absorbances of the OBP44a stock solutions were measured at 280 nm on an Agilent 8453 nanodrop instrument to characterize their concentrations based on the extinction coefficient predicted for OBP44a (11,710 M^−1^ cm^−1^). The stock concentrations were in the range of 600–1,200 μM. The ^15^N OBP44a and the OBP44a in complex with fatty acid were 200 μM concentration, whereas the ^13^C,^15^N samples used to acquired 3D NMR data for assignments were 400 μM. The 8(Z)-eicosenoic fatty acid, when present, was added from a stock solution in DMSO to a 1.2:1 ratio to the protein concentration. All NMR samples contain 10% D_2_O.

### NMR spectroscopy

All NMR data were acquired at 25 °C on a 600 MHz Bruker spectrometer equipped with a cryogenic probe, except for the 3D ^15^N-edited NOESY-HSQC (τ_mix_ = 80 ms) and ^13^C-CT-HSQC which were acquired on a 900 MHz Bruker spectrometer with a cryogenic probe. The following additional NMR experiments were carried out for backbone and Hα/ Hβ assignments: 3D HNCO, CBCA(CO)NH (Grzesiek and Bax 1993), HNCACB (Wittekind and Mueller 1993), C(CO)NH, H (CCO)NH (Grzesiek et al. 1993) and HBHA(CBCACO)NH (Grzesiek and Bax 1993).

The ^15^N transverse relaxation times were acquired with the following relaxation delays: 4, 20, 40, 64, 96, 112, 136, and 160 ms (Barbato et al. 1992; Farrow et al. 1994). A total number of 768 × 128 complex points and 69.120 ms × 91.2 ms acquisition times were acquired for the t_2_ and t_1_ dimensions, respectively. Twenty-four scans were acquired for each t_1_ point. The intensities from the eight 2D spectra were fit to a single exponential to obtain the T_2_ values. The experimental error was estimated using a Monte-Carlo method by adding the value of spectral noise to the intensities and refitting the exponential decay 100 times.

Chemical shift perturbations (CSP) were calculated using the following equation (Strickland et al. 2017):

$$CSP_{HN} = \surd [0.5(\left( {{\text{H}}_{holo - apo} } \right)^2 + \left( {\alpha \left( {N_{holo - apo} } \right)} \right)^2 )]$$

where α is a scaling factor calculated to be 0.12 and H_holo-apo_ describes the difference in amide ^1^H chemical shifts for the two forms and N_holo-apo_ describes the difference in amide ^15^N chemical shifts for the two forms.

All NMR data were processed using NMRPipe and analyzed with CcpNMR AnalysisAssign v3.1 (Skinner et al. 2016).

## Extent of assignments and data deposition

**Fig. 1:**
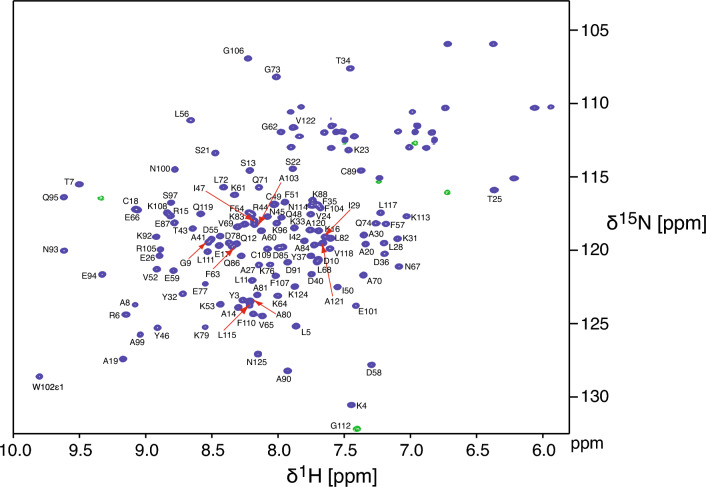
The backbone assignment of OBP44a bound to 8(Z)-eicosenoic acid. The 2D [^1^H-^15^N]-HSQC spectrum of the OBP44a in complex with the 8(Z)-eicosenoic acid was acquired at 600 MHz proton frequency and 25 °C. The resonance peaks are labeled with their assignments. Folded resonances in the indirect dimension are indicated by dashed contours and colored green

Backbone, Cβ and Hβ NMR resonance assignments for the 125 residue apo and holo OBP44a were completed. For apo OBP44a 121 of 122 expected backbone HN pairs were assignable with the exception of the N-terminal serine. Cα/Hα and Cβ/Hβ assignments were completed for 124 residues, including three prolines (P38, P98 and P116). Carbonyl carbons were assigned for all residues less the N-terminal Ser and the three residues preceding prolines (Y37, S97 and L115) as there is no amide proton available for the magnetization transfer required in the HNCO experiment. For OBP44a bound to 8(Z)-eicosenoic acid, assignments for 120 of 122 expected backbone amides were identified, with the exception of S1 and D2 (Fig. 1). Cα/Hα and Cβ/Hβ assignments were completed for 124 residues, including the three prolines (P38, P98 and P116). Carbonyl carbons were assigned for all residues except the N-terminal Ser and the three residues preceding prolines (Y37, S97 and L115). Chemical shift results for apo OBP44a and holo OBP44a are available under BMRB accession codes 52374 and 52377, respectively.

## Structural analysis

**Fig. 2:**
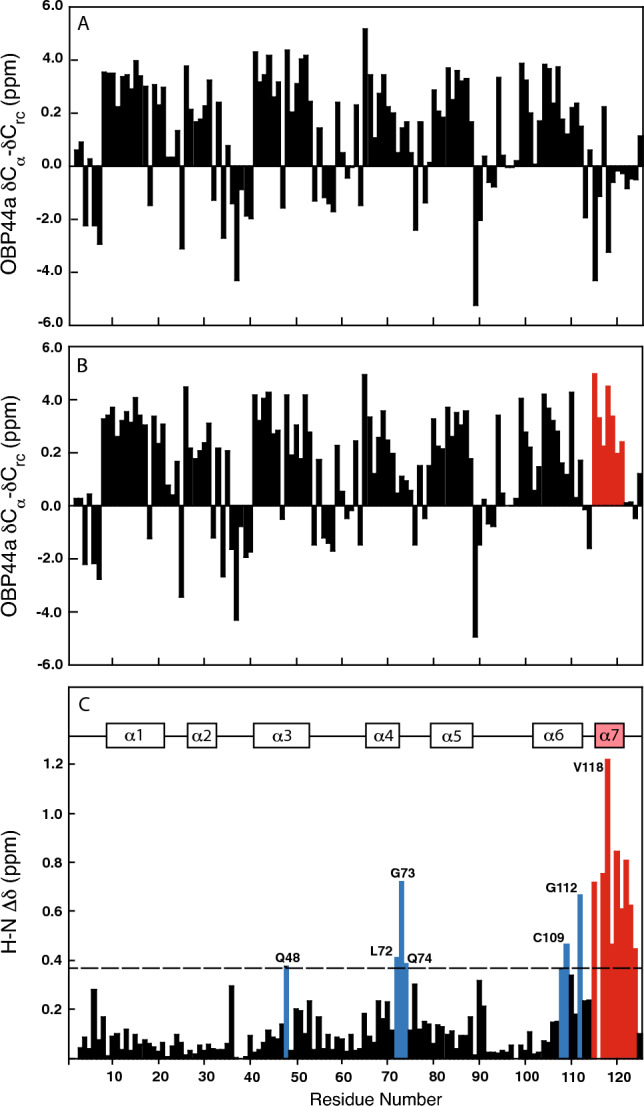
Secondary Cα carbon chemical shifts and chemical shift perturbation results showing that OBP44a undergoes a conformational change in the C-terminal tail upon binding 8(Z)-eicosenoic acid. **A** Cα secondary shifts for apo OBP44a indicate helical structure for six distinct regions. The first three helices have secondary shifts consistent with N-cap residues (T7, T25 and D40), while the 6th helix appears to be capped by a proline (P98). **B** Cα secondary shifts for holo OBP44a indicate helical structure for the same six stretches of amino acid sequence, but also indicate that a 7th helix (indicated by red bars) is formed upon binding with 8(Z)-eicosenoic acid. **C** ^1^H-^15^N chemical shift perturbations are shown upon OBP44a binding 8(Z)-eicosenoic acid. The dashed line represents one standard deviation (0.20) above the mean CSP for all residues (0.16), excluding prolines. Those residues with CSP larger than the cutoff and not in the C-terminal helix are shown in blue. Nine of the 16 residues (Q48, L72, G73, Q74, K108, C109, G112, L115, L117, V118, Q119, A120, A121, V122, Q123, and K124) experiencing significant changes in chemical shift reside in the predicted helix α7 and are shown in red

Chemical shifts of Cα were compared to random coil values (Spera and Bax 1991; Wishart et al. 1995) to determine secondary structure elements corresponding to six α-helices for apo OBP44a and seven α-helices for the holo form (Fig. 2A, B), all demonstrating uninterrupted stretches of downfield shifted Cα values. Examining secondary shifts for both Cα and Cβ, N-terminal helix capping residues were identified by a characteristic upfield shift of 1–2 ppm for Cα and a corresponding 1–4 ppm downfield shift of Cβ (Gronenborn and Clore 1994) at T7 (α1), T25 (α2), D40 (α3) for apo and holo OBP44a. As expected, backbone dihedral angle predictions for ϕ and ψ for each of these residues fall into known clusters for N-capping residues (Shen and Bax 2012). Helices α4 and α5 have charged residues where a capping residue would be expected (α4—K64, α5—D78), which might suggest salt-bridge stabilization of the helix termini. Helix α6 has a proline near its N-terminal end (P98); and though not flanked by the expected residues for a Pro-box motif, predicted backbone dihedral angles for 6 residues including the N-cap proline are consistent with those observed for the Pro-box motif (Viguera and Serrano 1999). Potential C-capping residues include G73 (helix α-4), and though not a conventional C-capping residue, the disulfide bond involving C89 likely stabilizes the end of helix α5. Helix α6 has a glycine in the C’ position, however, it does not meet the criteria of positive ϕ/ψ angles observed for recognized capping motifs.

Both Cα secondary shifts (Fig. 2B) and ^1^H-^15^N chemical shift perturbation (Fig. 2C) for holo OBP44a show the formation of a 7th helix upon binding eiconesoic acid. This binding-induced helix may be stabilized by the presence of an N-terminal asparagine (N114) in the N-cap position and/or the presence of a Pro-box motif (Viguera and Serrano 1999), as suggested by the amino acid sequence ^114^NLPLVQA. Additional data and structure determination are needed to clarify this. It does not appear to have a commonly recognized C-capping motif.

## Discussion

**Fig. 3.**
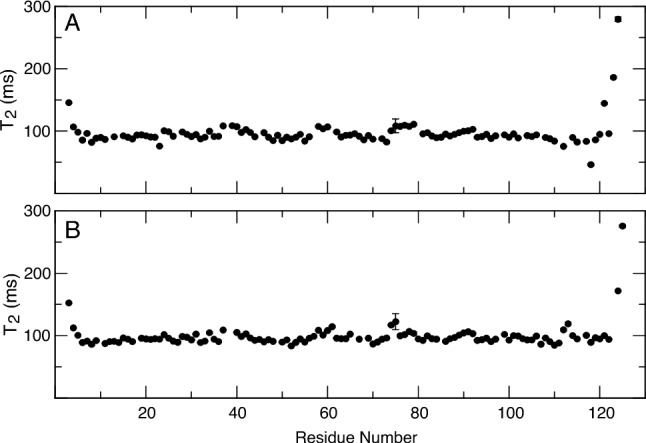
^15^N transverse relaxation of the free and 8(Z)-eicosenoic acid bound forms of OBP44a. The ^15^N transverse relaxation times were acquired at 600 MHz proton frequency and 25 °C. **A** The ^15^N T_2_ relaxation times for OBP44a are plotted against the residue number. **B** The relaxation times for protein bound to 8(Z)-eicosenoic acid are plotted as a function of residue number. The error bars indicate experimental error calculated from the noise in the spectra. There is very little variation in the T_2_ values in both forms of the protein, except at the termini. The binding of fatty acid modulates the relaxation times of the C-terminal residues. The C-terminal residues, except the terminal two, become more rigid as the protein binds the ligand

In summary﻿﻿, *Drosophila* OBP44a is a purely helical protein similar to the other members of the insect OBP family. It contains six α-helices in its ligand free form, with an additional α-helix forming upon ligand binding. In addition, OBP44a is a disulfide-containing protein like the other members of the family. It belongs to the C-Minus type subfamily, which features proteins with two disulfide bonds formed by four cysteines. Observed Cβ chemical shifts for all four cysteine residues in apo and holo OBP44a are shifted downfield relative to reduced values, confirming that they are in the oxidized state consistent with disulfide bond formation. Previous biochemistry experiments showed that urea in the absence of reducing agent was not enough to force the release of the ligand (He et al. 2023), further demonstrating that the disulfides stabilize the OBP44a fold. This stability is also reflected in the NMR relaxation profile, where the T_2_ indicates no significant chemical exchange or internal fast-motion contributions to the dynamics of any segment of the protein other than the extreme N- and C-terminal (Fig. 3).

Fatty acid binding,which is in slow exchange on the NMR timescale, involves transition of the C-terminus from random coil to α-helical. Similar to OBP22 (AeOBP22) from the salivary gland of *Anopheles gambiae*, OBP44a has a C-terminal helix that is expected to form a cap on the hydrophobic binding pocket (Jones et al. 2019; Wang et al. 2020). Chemical shift perturbation (CSP) calculations show that 16 residues exhibit shift changes of one standard deviation (0.20) or greater above the mean (0.16) upon binding of 8(Z)-eicosenoic acid. Nine of the 16 significantly perturbed residues (L115, L117-K124) reside within the predicted 7th helix (Fig. 2C). Other residues showing significant changes upon fatty acid binding near the C-terminus of helix α6 (K108, C109, G112), immediately after helix α4 (L72-Q74) and in the latter half of helix α3 (Q48). Interestingly, not only hydrophobic residues that are expected to form the binding pocket, but some charged residues are affected by fatty acid. An inference can be made, while the NMR assignment for the bound fatty acid is still being carried out, that the bound ligand must adopt a specific conformation, as it is the case within AeOBP22.

As highlighted above, the amino acids affected by ligand binding are different in OBP44a compared to AeOBP22. This may be a key determinant in how OBP44a responds to environmental conditions as it recognizes, transports, and releases its ligand. Further investigation into which physiological conditions are important for ligand binding in OBP44a might offer clues about how its structural features promotes specific biological roles in the development of the fly brain.

## Supplementary Information

Below is the link to the electronic supplementary material.

Supplementary file1 (DOCX 951 KB)

## Data Availability

The chemical shift assignments for the apo-OBP44a (BMRB 52374) and OBP44a-8(Z)-eicosenoic acid complex (BMRB 52377) have been deposited within the Biological Magnetic Resonance Data Bank.
