# Supplementary material for: NMR chemical shift assignment of Drosophila odorant binding protein 44a in complex with 8(Z)-eicosenoic acid
Source: Biomol NMR Assign. 2024 Jun 1;18(2):129–34. doi: 10.1007/s12104-024-10178-2 (PMC11511771; doi:10.1007/s12104-024-10178-2)
Supplement: Supplementary file 1 — Supplementary file1 (DOCX 951 KB) [file 12104_2024_10178_MOESM1_ESM.docx]

Biomolecular NMR Assignment

**NMR chemical shift assignment of *Drosophila* odorant binding protein 44a in complex with 8(Z)-eicosenoic acid**

Myriam L. Cotten^1#^, Mary R. Starich^2#^, Yi He^3^, Jun Yin^4^, Quan Yuan^4^, and Nico Tjandra^2^*

*^1^ Department of Biochemistry and Biophysics, Oregon State University, Corvallis, OR 97331*

*^2^ Laboratory of Molecular Biophysics, Biochemistry and Biophysics Center, National Heart, Lung, and Blood Institute, National Institutes of Health, Bethesda, MD 20892*

*^3^ Fermentation Facility, Biochemistry and Biophysics Center, National Heart, Lung, and Blood Institute, National Institutes of Health, Bethesda, MD 20892*

*^4^ Dendrite Morphogenesis and Plasticity Unit, National Institute of Neurological Disorders and Stroke, National Institutes of Health, Bethesda, MD 20892*

*^#^Equal contribution*

**Corresponding author: Nico Tjandra*

*E-mail address:* [*tjandran@nhlbi.nih.gov*](mailto:tjandran@nhlbi.nih.gov)

*Phone: (301)-402-3029*

*Fax: (301)-402-3405*

**Keywords:** odorant binding protein, fatty acid binding protein, lipid interaction

**Fig. S1.** An overlay of the 2D [^1^H-^15^N]-HSQC spectra of the OBP44a free and in complex with the 8(Z)-eicosenoic acid that were acquired at 600 MHz proton frequency and 25°C. The resonances of the complex of OBP44a and the fatty acid are in blue, with folded resonances in dash contours and colored green. The resonances of OBP44a free of fatty acid are in red, while folded peaks are in dashed contour and colored orange. The C-terminal residues that show large chemical shift changes are labeled in red and blue for the apo- and holo-OBP44a, respectively.
